# Serum Heparin-Binding Protein as a Potential Biomarker to Distinguish Adult-Onset Still’s Disease From Sepsis

**DOI:** 10.3389/fimmu.2021.654811

**Published:** 2021-03-31

**Authors:** Rui Tian, Xia Chen, Chengde Yang, Jialin Teng, Hongping Qu, Hong-Lei Liu

**Affiliations:** ^1^ Department of Critical Care Medicine, Ruijin Hospital, Shanghai Jiao Tong University School of Medicine, Shanghai, China; ^2^ Department of Rheumatology and Immunology, Ruijin Hospital, Shanghai JiaoTong University School of Medicine, Shanghai, China

**Keywords:** adult-onset Still’s disease (AOSD), sepsis, heparin-binding protein (HBP), biomarker, autoinflammatory diseases

## Abstract

Adult-onset Still’s disease (AOSD) is a systemic, multifactorial, autoinflammatory disease for which the etiopathogenesis is not well understood. Given the similarities in clinical and laboratory features between this disease and sepsis, and the differences in treatment strategies for these two diseases, specific diagnostic markers are crucial for the correct diagnosis and management of AOSD. Previous studies have shown plasma heparin-binding protein (HBP) is a promising potential biomarker for AOSD; thus, this study aimed to detect serum HBP levels in patients with AOSD or sepsis to assess its potential as a biomarker for differential diagnosis. We found that serum HBP levels were significantly higher in patients with active AOSD than that in those with inactive AOSD. Patients with sepsis had higher serum HBP levels compared with those who had active or inactive AOSD. We calculated the area under the receiver operating characteristic (ROC) curve to assess whether HBP could be used to differentiate active from inactive AOSD; this was 0.811 with sensitivity 0.650, specificity 0.811, and cutoff HBP value of 35.59 ng/ml. The area under the ROC curve for HBP as a biomarker to differentiate AOSD from sepsis was 0.653, with sensitivity 0.759, and specificity 0.552, and cutoff HBP value of 65.1 ng/ml. Taken together, the results of our study suggest that serum HBP could be a useful diagnostic biomarker to evaluate disease activity in patients with AOSD, and to differentiate AOSD from sepsis.

## Introduction

Adult-onset Still’s disease (AOSD) is a rare, systemic autoinflammatory disorder, typically characterized by spiking fever, arthritis or arthralgia, rash and increased serum ferritin; other frequently observed clinical features include a sore throat, enlargement of the liver and spleen, and swelling of the lymph nodes (1–3).

Chronic AOSD occurs in episodes of activity, or ‘flares’; during a disease flare, laboratory tests often show raised erythrocyte sedimentation rates and C-reactive protein levels, along with high levels of ferritin (4). However, currently, there are no established diagnostic criteria for AOSD. In clinical treatment, this disease is typically diagnosed according to clinical characteristics and laboratory parameters after acute infection (especially sepsis), malignancy and other autoimmune or autoinflammatory diseases have been ruled out (5). However, in clinical practice, differentiating AOSD from sepsis remains challenging. Sepsis is a life-threatening complex condition that results in a dysregulated host response to severe infection, acute organ dysfunction, and high morbidity and mortality in hospitals (6); clinical characteristics and symptoms overlap with AOSD, and therefore in some cases it is difficult to differentiate between these conditions. However, the treatment plans for AOSD and sepsis are very different: sepsis may be treated with antimicrobial agents, while AOSD may require immunosuppressive therapy, which can reduce immune resistance and cause or aggravate infection in sepsis patients (7). Early identification of AOSD is therefore vital for efficient and successful clinical management of this disease.

Several studies have made progress in distinguishing the two diseases clinically in their early phases, but few potential biomarkers for AOSD with sufficiently high sensitivity and specificity have been identified. Previous studies indicate that levels of C-reactive protein, procalcitonin, lactate, some cytokines such as interleukin 18 (IL-18) and white blood cells (8–12) could help to distinguish between AOSD and sepsis. However, there were some reviews or meta-analysis indicated that these potential indicators have limited specificity in clinical practice (8, 13, 14).

Recently, a prospective cohort study in China (15) found that serum heparin-binding protein (HBP) levels in patients with sepsis were significantly higher than in those with localized infections; HBP also had a higher value for the area under the receiver operating characteristic (ROC) curve than other parameters currently used for identifying sepsis (14). HBP is released by neutrophils in the blood as part of the innate immune response, which is the first line of defense against bacteria or other pathogens (16). Studies have shown that HBP is the key mediator of neutrophil-induced vascular endothelial permeability, which can amplify systemic inflammatory responses by inducing cytoskeletal rearrangement and resulting in vascularity and neutrophil exosmosis and aggregation (17, 18). Plus, HBP could be an early biomarker to evaluate the severity of sepsis (18). HBP may also be positively correlated with C-reactive protein and procalcitonin, other indicators of inflammation, in inflammatory diseases (19). Recently, in a study of people with symptoms of coronavirus (COVID-19), some of those with a rheumatologic background also had high serum HBP levels, though they were not ultimately diagnosed with a viral infection or sepsis; this indicates that high HBP levels may be associated with autoinflammatory diseases.

HBP (also called azurocidin, cationic protein or CAP37), with a molecular weight of 37kDa, is a serine protease derived from polymorphonuclear neutrophils (20). Serum HBP is an established diagnostic marker of sepsis (19, 21–24), circulatory failure (19), acute kidney injury (25–28), acute lung injury/acute respiratory distress syndrome (26, 29, 30) and other infectious diseases (31). However, until now, no research has investigated whether HBP levels could contribute to the identification of autoinflammatory diseases such as AOSD. Thus, we compared serum HBP levels in a cohort of patients with AOSD and those with sepsis to determine whether HBP could be used as a biomarker to differentiate between these conditions; we also explored whether HBP levels could be used to distinguish between active and inactive disease states in AOSD.

## Materials and Methods

### Study Design and Recruitment

Patients with AOSD diagnosed according to the Yamaguchi criteria (5), those with sepsis or septic shock according to the Sepsis-3 definitions (32), and a control group of healthy individuals (without history of chronic diseases such as diabetes, hypertension and heart disease; normal parameters of complete blood count and biochemical panel) were recruited from Ruijin Hospital (Shanghai, China). Patient characteristics (such as age and gender), medical histories (mainly symptoms and signs at the time of onset or remission) and laboratory testing results (such as a complete blood count, liver and renal function tests, CRP and ferritin) were reviewed. The aim of this study was to determine the correlation of serum HBP level with disease activity and laboratory parameters in AOSD patients and to explore its possible role as a new biomarker for differential diagnosis between AOSD and sepsis.

AOSD disease activity was assessed according to the modified Pouchot score proposed by Rau and colleagues (33), which takes into account the presence or absence of each of the following 12 clinical features: fever, rash, sore throat, arthritis/arthralgia, myalgia, pleuritis, pericarditis, pneumonitis, lymphadenopathy (enlarged lymph glands), hepatomegaly (enlarged liver) or abnormal liver function (elevated liver enzymes), leukocyte count > 15,000/μl, and serum ferritin > 3000 μg/L.

Patients with AOSD in this study were included in the ‘active’ AOSD subgroup if they had not received any treatment for their condition (treatment-naïve) at the onset of the disease and met two or more of the following diagnostic criteria of AOSD: spiking fever, inflammatory arthralgia/arthritis, transient rash, and pharyngitis or sore throat. If they did not meet these criteria, and AOSD-related histopathological tests had been normal for at least two consecutive months, they were included in the ‘inactive’ subgroup of patients with the disease (34, 35).

### Ethical Approvals and Patient Consent

This study was approved by the Institutional Research Ethics Committee of Ruijin Hospital (2016-62), Shanghai, China.

### Blood Samples

Venous blood samples for the measurement of HBP were obtained. All blood samples were spun in a centrifuge at 3000 revolutions per minute at 4°C for 10 minutes. Serum levels of HBP were measured in an enzyme-linked immunosorbent assay (ELISA) using a commercially available ELISA kit (Joinstar Biomedical Technology Co., LTD, China, HBP20S1A02F) according to the manufacturer’s standard protocol using a Jet-iStar 3000 rapid immunoassay analyzer.

### Statistical Analysis

All statistical analyses were performed using the IBM SPSS statistics package (version 23.0). Figures were prepared using GraphPad Prism version 6.0 (GraphPad Software). Continuous data were presented as mean ± standard error of mean (SEM) or mean with standard deviation (SD), and categorical data were presented as absolute numbers and percentages. As all data were normally or approximately normally distributed, we used Person correlation analysis and one-way analysis of variance (ANOVA) to explore between-variable relationships. We used Spearman correlation to analyze the correlation between Pouchot score and HBP. Correlation between HBP level and clinical disease manifestations was calculated using two-tailed Student’s t-test. The receiver operating characteristic (ROC) curve was used to assess the predictive value of HBP. All analyses were exploratory; thus, a two‐tailed p‐value of < 0.05 was used to assess statistical significance.

## Results

### Patient Characteristics

In total, 30 patients with AOSD, 29 patients with sepsis, and 30 healthy individuals were enrolled in the study. Of those with AOSD, 20 had active and 10 had inactive disease at the time their blood sample was taken, according to Rau’s criteria (33). Laboratory blood test results for those included in our study are shown in **Table 1**.

**Table 1:** Characteristics of people with adult-onset Still’s disease (AOSD) and those with sepsis and healthy controls included in this study.

Characteristics	Active AOSD	Inactive AOSD	Sepsis	HC
Age, mean (range)	34.8(19-59)	35.1(22-56)	57.6(19-80)	31.8(20-54)
Gender (female/male)	15/5	9/1	23/6	23/7
Laboratory values, mean ± SEM				
WBC (10^9^/L)	13.9 ± 2.0	10.9 ± 1.2	14.7 ± 2.1	/
N (10^9^/L)	11.0 ± 2.0	8.0 ± 1.1	11.7 ± 1.5	/
Platelets (10^9^/L)	274.7 ± 37.8	248.0 ± 14.1	172.9 ± 19.7	/
Lymphocyte (10^9^/L)	2.2 ± 0.4	/	0.9 ± 0.1	/
Hemoglobin (g/L)	108.1 ± 4.2	133.0 ± 5.0	88.9 ± 3.8	/
ALT (IU/L)	187.8 ± 115.9	17.5 ± 3.3	49.0 ± 9.7	/
AST (IU/L)	82.3 ± 28.6	16.0 ± 1.0	67.6 ± 15.0	/
Creatinine (umol/L)	56.8 ± 3.3	77.1 ± 8.9	151.9 ± 32.9	/
Urea (mmol/L)	4.1 ± 0.4	/	11.7 ± 1.7	/
CRP (mg/L)	37.7 ± 14.8	1.7 ± 0.6	149.8 ± 18.3	/
PCT (ng/ml)	/	/	27.1 ± 9.8	/
ESR (mm/h)	42.5 ± 8.3	8.1 ± 1.8	/	/
Lactate (mmol/L)	/	/	3.2 ± 0.5	/
Ferritin (ng/ml)	4587.1 ± 1555.6	82.1 ± 28.2	/	/
AOSD Parameters				
Fever	15(75.0%)	0(0)	/	/
Sore throat	16(80.0%)	0(0)	/	/
Evanescent rash	14(70.0%)	0(0)	/	/
Arthralgia	15(75.0%)	0(0)	/	/
Pneumonia	3(15.0%)	3(30.0%)	/	/
Pericarditis	1(5.0%)	0(0)	/	/
Hepatomegaly	1(5.0%)	1(10.0%)	/	/
Splenomegaly	6(30.0%)	2(20.0%)	/	/
Lymphadenopathy	11(55%)	3(30.0%)	/	/
Myalgia	9(45.0%)	2(20.0%)		/
Pleurisy	3(15.0%)	0(0)	/	/
Weight loss	4(20.0%)	1(10.0%)	/	/
Stomach ache	1(5.0%)	0(0)	/	/
Renal dysfunction	0(0)	0(0)	/	/
Site of infection, n (%)				
Lung	/	/	7(24.1%)	/
Abdominal	/	/	22(75.9%)	/
Blood	/	/	12(41.4%)	/
Others	/	/	6(20.7%)	/
28-day mortality, n (%)	/	/	2(6.7%)	/

Data are presented as n (percentages), mean ± SEM

APACHE II, Acute Physiology and Chronic Health Evaluation II; CRP, C‐reactive protein; n, numbers; SEM, mean ± standard error of mean; SOFA, Sequential Organ Failure Assessment; WBC, white blood cell count.

### Serum HBP Levels in People With AOSD Versus Those With Sepsis

**Figure 1:**
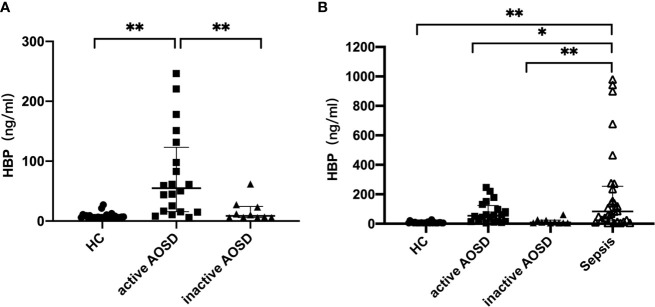
Comparative analysis of HBP in patients with active AOSD patients, inactive AOSD patients, healthy controls **(A)** and Sepsis **(B)**. Concentrations of HBP in serum form HC (healthy controls, filled circles), active AOSD patients (filled squares), inactive AOSD patients (filled triangles) and Sepsis patients (inversed hollow triangles). Data are presented as mean with standard deviation (SD). *P < 0.05, **P < 0.01.

Serum HBP levels in patients with active AOSD, inactive AOSD, and those with sepsis are reported in **Table 2**. The level of HBP in people with active AOSD was significantly higher than in the inactive AOSD group and in healthy controls (p < 0.05; **Figure 1A**); there was no significant difference in HBP levels between the inactive AOSD and healthy control groups, as shown in **Figure 1A**. Serum HBP in people with sepsis was significantly higher than in those with active or inactive AOSD and in healthy controls (p < 0.05; **Figure 1B**).

**Table 2:** Serum heparin-binding protein (HBP) levels in patients with adult-onset Still’s disease (AOSD), those with sepsis, and healthy control individuals.

VariableMean(SD)	Healthy Controls	AOSD	Sepsis
HBP (ng/ml)	8.3089(4.9153)	56.414(66.1662)	204.7045(295.1682)

### Correlation of HBP Levels With Other Blood Test Results and Disease Activity Score

**Figure 2:**
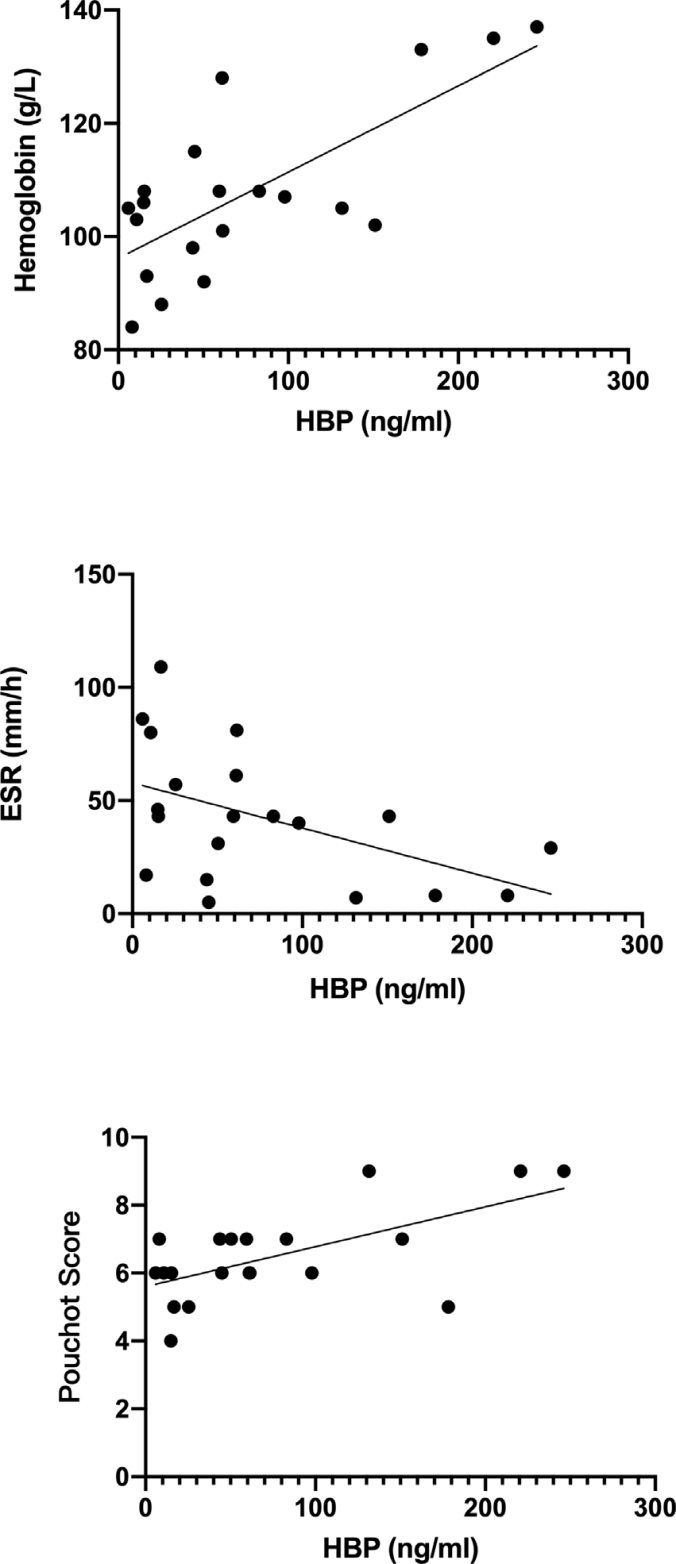
Correlations between heparin-binding protein (HBP) and other laboratory indicators in blood taken from patients with active adult-onset Still’s disease.

Correlations between HBP and laboratory values of all active AOSD patients are shown in **Figure 2**. Notably, HBP was significantly positively correlated with hemoglobin level (r = 0.732, p < 0.05) and negatively correlated with erythrocyte sedimentation rate (r = –0.491, p < 0.05); HBP was also significantly positively correlated with the modified Pouchot disease activity score (r =0.469, p =0.037).

### Correlation Between HBP Level and Clinical Disease Manifestations in AOSD

**Figure 3:**
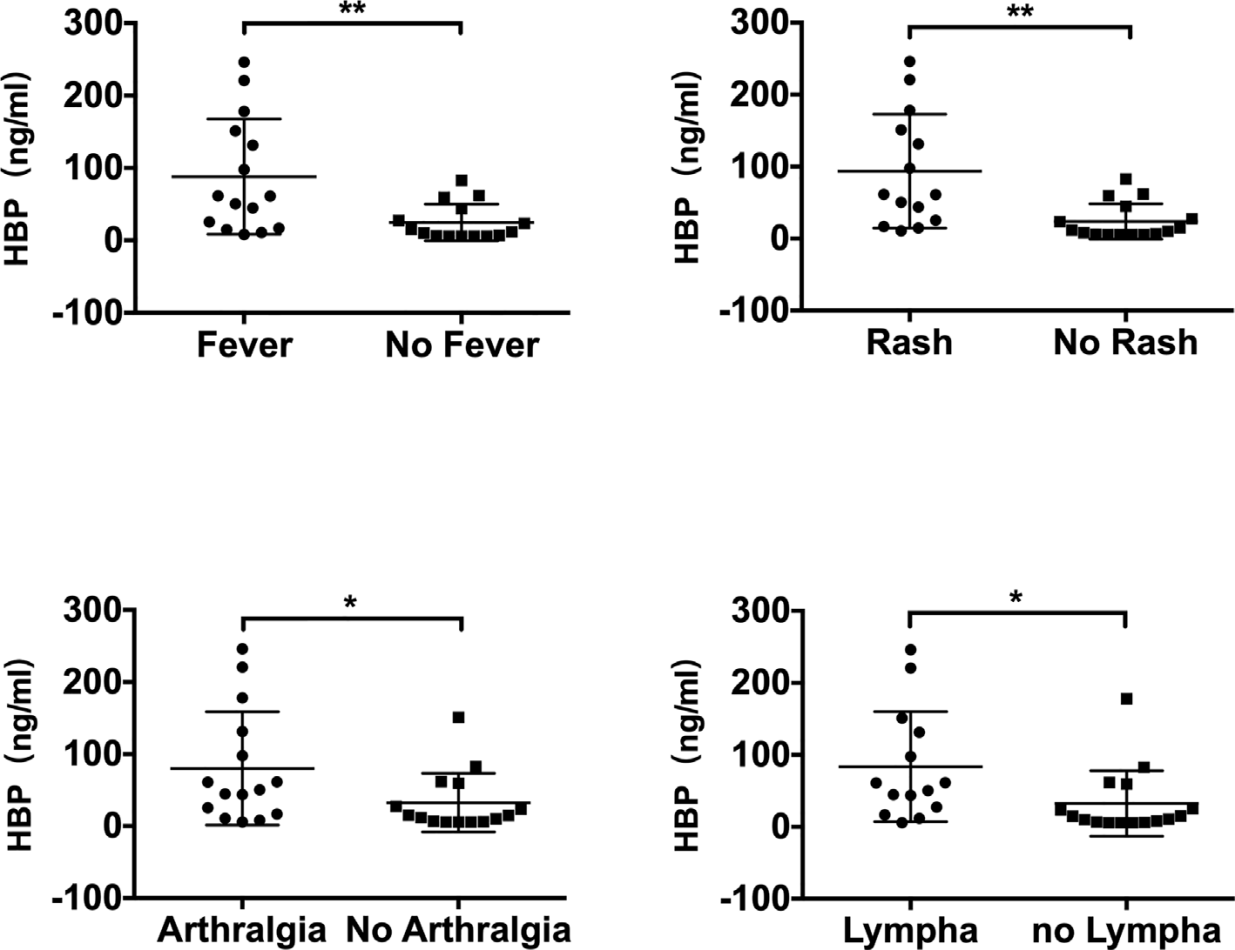
Comparison of serum HBP levels in patients with adult-onset Still’s disease with different measures of clinical disease manifestation (“lympha” is short for “lymphadenopathy”). Data are presented as mean and interquartile range and significance was calculated using two-tailed Student’s t-test. *P < 0.05, **P < 0.01.

The HBP level was found to be significantly higher in patients with active AOSD who had arthralgia (p=0.047), fever (p=0.007), rash (p=0.002), or lymphadenopathy (abbreviated as lympha in **Figure 3**) (p=0.032), as shown in **Figure 3**. Univariate logistic regression analysis (**Table 3**) was performed to determine the predictive power of fever, rash, arthralgia and lymphadenopathy. The variables showed significant predictive value within univariate analysis and were included in further stepwise multivariate logistic regression (**Table 3**). The regression analysis detected rash (OR 0.535, P = 0.002) as an independent significant predictor of HBP in patients with active AOSD.

**Table 3:** Univariate and multivariate linear analysis of clinical disease manifestations in AOSD.

Variables	Univariate linear analysis		Multivariate linear analysis	
	OR	P	OR	P
Fever	0.439	0.015*	/	/
Rash	0.535	0.002*	0.535	0.002*
Arthralgia	0.366	0.047*	/	/
Lymphadenopathy	0.392	0.032*	/	/

*P < 0.05.

### Determination of HBP Cutoff Level to Determine AOSD

**Figure 4:**
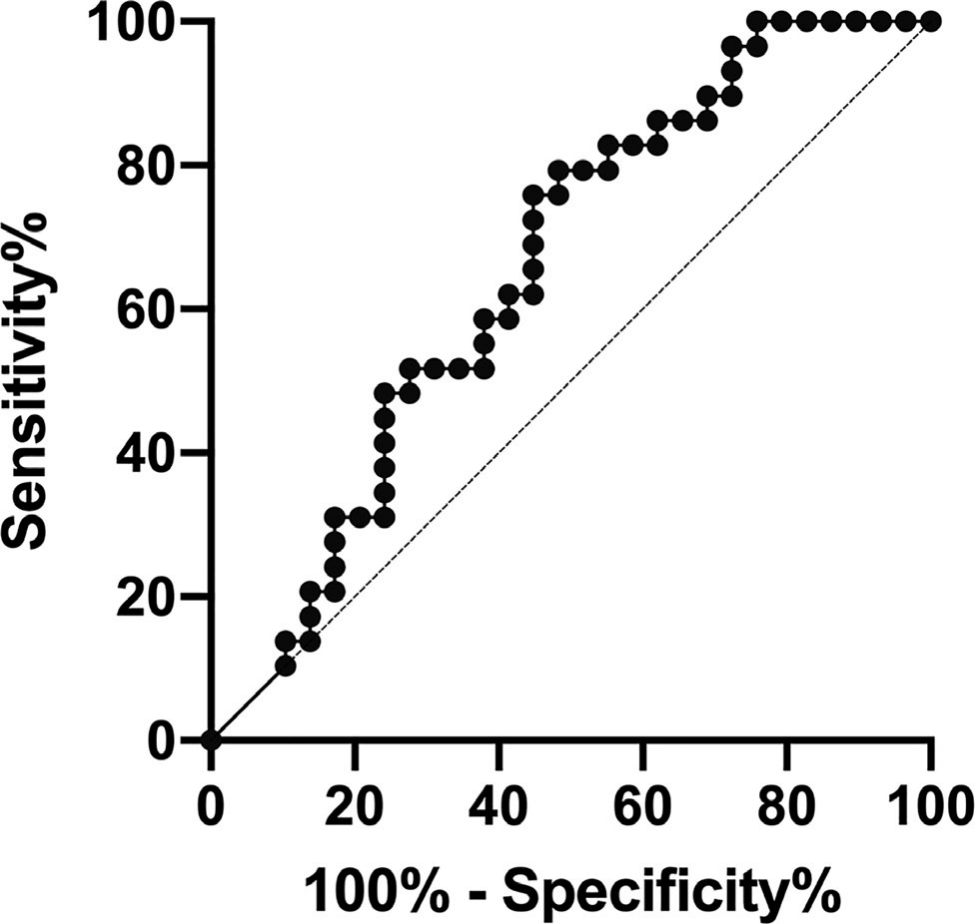
Receiver operating characteristic (ROC) curve analysis of the predictive capacity of heparin-binding protein (HBP) to distinguish between adult-onset Still’s disease (AOSD) and sepsis.

The area under the ROC curves used to determine whether HBP could be used to differentiate AOSD from sepsis was 0.653 (sensitivity, 0.759; specificity, 0.552; p < 0.05; **Table 4**, **Figure 4**). The cutoff HBP level was 65.1 ng/ml. In the ROC curves used to assess the predictive capacity of serum HBP for active versus inactive AOSD, the value for the area under the ROC curve was high (0.811; sensitivity, 0.650; specificity, 0.889; p < 0.01, **Table 4**). The cutoff HBP level was 35.59 ng/ml. These results demonstrate that the serum HBP is a potentially useful biomarker to distinguish active AOSD from inactive disease and from sepsis.

**Table 4:** Receiver operating characteristic (ROC) curve analysis of the predictive capacity of heparin-binding protein (HBP) to distinguish between active and inactive AOSD and sepsis.

Variable	AUC ROC	95%CI	P-value	Sensitivity(%)	Specificity(%)	Cut-off(ng/ml)
Active AOSD *vs.* Inactive AOSD	0.811	0.644-0.979	0.008*	0.650	0.889	35.59
AOSD *vs.* Sepsis	0.653	0.511- 0.796	0.045*	0.759	0.552	65.1

*P < 0.05.

## Discussion

Early differentiation between infections such as sepsis and many systemic autoinflammatory disorders including AOSD is a significant challenge in clinical practice. In this study, for the first time, we showed that serum HBP could be a useful biomarker to distinguish sepsis from AOSD and to determine disease activity in patients with AOSD.

Several biomarkers have been proposed to distinguish the two diseases in early phase (8–12), and in particular, studies have showed that serum IL-18 could be used as a biomarker for differential diagnosis and AOSD disease evaluation (35). Serum IL-18 levels have been shown to be significantly higher in patients with AOSD than in other inflammatory diseases, and its concentration correlates with disease activity. IL-18 may therefore be a useful therapeutic target for AOSD as well as a biomarker for differential diagnosis between AOSD and sepsis (35–39). Until now, there were no other biomarkers with high sensitivity and specificity.

In our study, we found that the level of serum HBP was higher in patients with sepsis compared with those with active AOSD; the cutoff value was 65.10 ng/ml, with higher levels indicating sepsis, and lower levels indicating AOSD. Furthermore, we identified that the serum HBP level was higher in active AOSD patients compared with inactive AOSD patients; our ROC analysis showed that HBP had a high value for the area under the ROC curve in active AOSD patients, with a high sensitivity and specificity. Our results demonstrate that HBP could be a useful, novel biomarker that could be used as an alternative or in conjunction with IL-18 for improved diagnostic accuracy to distinguish between sepsis and AOSD, as well as active and inactive AOSD. This, in turn, could improve early diagnosis and effective treatment for both conditions.

HBP is prefabricated in neutrophils and stored in azure granules and secretory vesicles, and can be released immediately following stimulation by substances produced by bacteria, inflammatory cytokines, and chemotactic factors, leading to increased blood vessel permeability and edema (16, 18). HBP is also a powerful chemoattractant for a variety of innate immune cells, primarily monocytes and macrophages (19). These characteristics may partly explain the strong association between serum HBP levels and inflammatory processes in active AOSD. Our study also showed that serum HBP had a positively correlation with hemoglobin levels, and a negative correlation with erythrocyte sedimentation rate. Further research on the possible mechanisms by which HBP regulates disease activity in AOSD, and to what extent HBP can influence the level of hemoglobin in the blood, is needed. Moreover, our study also found that the number of patients who had fever and rash are almost the same. A large retrospective study by Liu and his colleagues found that the incidence of skin rash was 79.9% among Chinese active AOSD patients (40). Yang et al. found that the incidence of rash was almost 88.5% among in a Chinese cohort study (41). As AOSD typically manifests with a symptomatic triad characterized by spiking fever, arthritis and rash, coupled with our small number of samples, it may lead to a high overlap rate of these two symptoms.

Our study has several limitations that should be acknowledged. First, as AOSD is a rare condition, the number of AOSD patients included in this study is relatively small which may result in bias. Second, the study was performed as a single-center study, and future studies with larger cohorts should be performed to validate these results and to reveal a dynamic role for discriminating AOSD from sepsis in clinical practice. Third, several biomarkers such as CRP, ferritin, IL-18 and IL-37 were proposed as potential parameters for assessing disease activity status of AOSD (42–44); Rau et al. described a systemic scoring system comprised of 12 main signs and symptoms (33). However, no exact and reliable criteria exist yet. We define AOSD disease clinically active or inactive based on our understanding of this disease in our clinical work (34, 35, 44–47). Grouping AOSD disease activity status by our method was somewhat arbitrary and divergent, so it may cause the results to be biased with other research units. Last, previous studies had proposed that PCT, IL-18 combined with fibroblast growth factor 2 (FGF 2) and modified Pouchot Score including elevated serum ferritin levels (12, 33, 36) could be a useful diagnostic tool to distinguish AOSD and sepsis. However, as our study was a retrospective design, many laboratory values such as ferritin, PCT, serum cytokine levels such as IL-18, IL-6 and interferon were not performed in all patients at that time. Thus, it was hard for us to verify and make the discrimination by these indicators. To overcome these issues, future studies with larger cohorts and more comprehensive serological tests should be performed to validate these results and to reveal a dynamic role for discriminating AOSD from sepsis in clinical practice.

In conclusion, our study indicates that serum HBP could be used as a diagnostic biomarker to differentiate sepsis from AOSD when the two conditions may otherwise be indistinguishable, and to determine disease activity in patients with AOSD. Further studies are required to confirm these results and establish their use in clinical practice.

## Data Availability Statement

The raw data supporting the conclusions of this article will be made available by the authors, without undue reservation.

## Ethics Statement

The studies involving human participants were reviewed and approved by Institutional Research Ethics Committee of Ruijin Hospital (identifier 2016–62). The patients/participants provided their written informed consent to participate in this study.

## Author Contributions

RT and XC: contributed equally to this work. H-LL, CY, JT, HQ: conceived and designed the experiments. RT and XC: performed the experiments. RT: analysed the data. XC: contributed reagents/materials/analysis tools. CY and JT: supervised the study. RT and XC: wrote the article. All authors contributed to the article and approved the submitted version.

## Funding

This research was funded by National Natural Science Foundation of China (81502016 and 81772040) and Shanghai Municipal Health and Family Planning Commission General Hospital Chinese and Western Medicine Clinical Cooperation Pilot Construction Project (ZY (2018-2020)-FWTX-1108).

## Conflict of Interest

The authors declare that the research was conducted in the absence of any commercial or financial relationships that could be construed as a potential conflict of interest.
